# *N*,*N*′-{[Ethane-1,2-diylbis(­oxy)]bis­(ethane-2,1-di­yl)}bis­(4-methyl­benzene­sulfonamide)

**DOI:** 10.1107/S1600536812024324

**Published:** 2012-06-02

**Authors:** Nassir N. Al-Mohammed, Yatimah Alias, Zanariah Abdullah, Hamid Khaledi

**Affiliations:** a Department of Chemistry, University of Malaya, 50603 Kuala Lumpur, Malaysia

## Abstract

The asymmetric unit of the title compound, C_20_H_28_N_2_O_6_S_2_, contains one half-mol­ecule, related to the other half by a twofold rotation axis. The two aromatic rings of the mol­ecule make a dihedral angle of 50.91 (7)°. The O—CH_2_—CH_2_—O and N—CH_2_—CH_2_—O fragments both adopt *gauche* conformations, with torsion angles of 76.0 (4) and 70.4 (3)°, respectively. In the crystal, adjacent mol­ecules are linked through N—H⋯O hydrogen bonds into chains along the *a*-axis direction. The chains are further connected *via* C—H⋯O inter­actions into a two-dimensional supra­molecular network in the *ac* plane.

## Related literature

For similar structures, see: Polyakova *et al.* (1990 ▶); Ding *et al.* (2003 ▶).
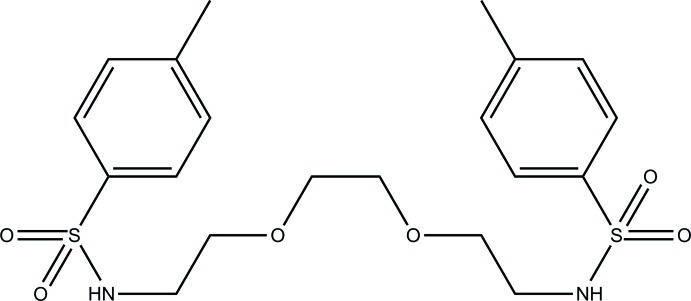


## Experimental

### 

#### Crystal data

C_20_H_28_N_2_O_6_S_2_
*M* *_r_* = 456.56
Monoclinic, 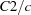

*a* = 11.135 (7) Å
*b* = 9.220 (6) Å
*c* = 21.452 (15) Å
β = 93.680 (12)°
*V* = 2198 (3) Å^3^
*Z* = 4
Mo *K*α radiation
μ = 0.28 mm^−1^
*T* = 296 K
0.23 × 0.14 × 0.04 mm

#### Data collection

Bruker APEXII CCD diffractometer
Absorption correction: multi-scan (*SADABS*; Sheldrick, 1996 ▶) *T*_min_ = 0.938, *T*_max_ = 0.989
5135 measured reflections
1983 independent reflections
1558 reflections with *I* > 2σ(*I*)
*R*_int_ = 0.055

#### Refinement

*R*[*F* ^2^ > 2σ(*F* ^2^)] = 0.053
*wR*(*F* ^2^) = 0.150
*S* = 1.03
1983 reflections
140 parameters
1 restraint
H atoms treated by a mixture of independent and constrained refinement
Δρ_max_ = 0.32 e Å^−3^
Δρ_min_ = −0.36 e Å^−3^

### 

Data collection: *APEX2* (Bruker, 2007 ▶); cell refinement: *SAINT* (Bruker, 2007 ▶); data reduction: *SAINT*; program(s) used to solve structure: *SHELXS97* (Sheldrick, 2008 ▶); program(s) used to refine structure: *SHELXL97* (Sheldrick, 2008 ▶); molecular graphics: *X-SEED* (Barbour, 2001 ▶); software used to prepare material for publication: *SHELXL97* and *publCIF* (Westrip, 2010 ▶).

## Supplementary Material

Crystal structure: contains datablock(s) I, global. DOI: 10.1107/S1600536812024324/pv2551sup1.cif

Structure factors: contains datablock(s) I. DOI: 10.1107/S1600536812024324/pv2551Isup2.hkl

Supplementary material file. DOI: 10.1107/S1600536812024324/pv2551Isup3.cml

Additional supplementary materials:  crystallographic information; 3D view; checkCIF report

## Figures and Tables

**Table 1:** Hydrogen-bond geometry (Å, °)

*D*—H⋯*A*	*D*—H	H⋯*A*	*D*⋯*A*	*D*—H⋯*A*
N1—H1⋯O2^i^	0.82 (2)	2.14 (2)	2.944 (3)	171 (3)
C6—H6⋯O1^ii^	0.93	2.56	3.311 (4)	138

Symmetry codes: (i) 
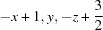
; (ii) 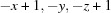.

**Fig. 1.**
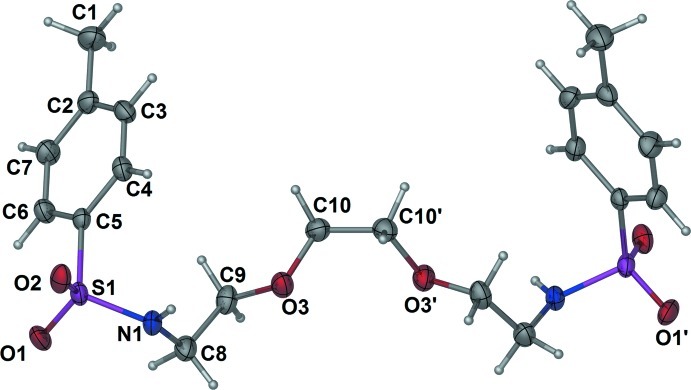
Molecular structure of the title compound with displacement ellipsoids drawn at 30% probability level. Hydrogen atoms are drawn as spheres of arbitrary radius. Symmetry code: ' = -x + 2, y, -z + 3/2.

**Fig. 2.**
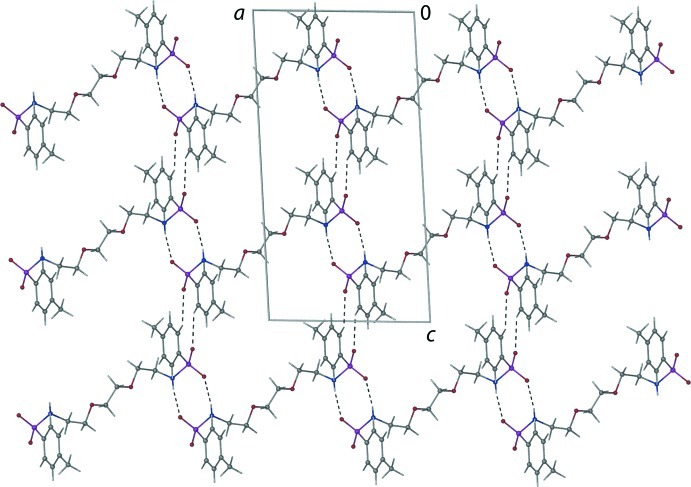
The 2-D array in the ac plane formed by N—H···O and C—H···O hydrogen bonds, depicted as dashed lines.

## supplementary crystallographic information

### Comment

The title compound (Fig. 1) was obtained through the condensation reaction of 1,8-diamino-3,6-dioxaoctane with *p*-toluenesulfonyl chloride. A twofold rotation axis passes through the mid-point of C10—C10' bond (symmetry code: ' = -*x* + 2, *y*, -*z* + 3/2), the asymmetric unit therefore comprises one half of the molecule. The two symmetry related aromatic rings in the molecule make a dihedral angle of 50.91 (7)°. Similar to what was observed in a related structure (Polyakova *et al.*, 1990), the O—CH_2_—CH_2_—O and N—CH_2_—CH_2_—O fragments adopt the *gauche* conformations with torsion angles of 76.0 (4) and 70.4 (3)° respectively. The S—O bond distances [1.423 (2) and 1.433 (2) Å] and S—N bond distance [1.608 (2) Å] are comparable to the values found in the literature (Ding *et al.*, 2003; Polyakova *et al.*, 1990). The crystal packing of the molecule shows layers in the *ac* plane formed by N—H···O and C—H···O interactions (Table 1, Fig. 2)

### Experimental

A solution of *p*-toluenesulfonyl chloride (2.83 g, 1.48 mmol) in dry dichloromethane (25 ml) was added drop wise to a dichloromethane solution (25 ml) of 1,8-diamino-3,6-dioxaoctane (1 g, 0.675 mmol) and triethylamine (2.34 ml, 1.69 mmol) at 273 K. The mixture was stirred at room temperature overnight, washed with water and saturated solution of NaHCO_3_ (3 *x* 10 ml) and dried over MgSO_4_. The organic layer was evaporated and the residue was dissolved in methanol. The colorless crystals of the title compound were obtained through slow evaporation of the methanolic solution at room temperature (m.p. = 361–363 K).

### Refinement

C-bound hydrogen atoms were located at the calculated positions and refined in riding mode with C—H distances of 0.93 (aryl), 0.96 (methyl) and 0.97 (methylene) Å. The amino hydrogen atom was found in a difference Fourier map and refined with a distance restraint of N—H 0.86 (2) Å. For H atoms, *U*_iso_(H) were set to 1.2 (1.5 for methyl) *U*_eq_(carrier atoms).

### Crystal data

**Table:**

C_20_H_28_N_2_O_6_S_2_	*F*(000) = 968
*M**_r_* = 456.56	*D*_x_ = 1.380 Mg m^−^^3^
Monoclinic, *C*2/*c*	Mo *K*α radiation, λ = 0.71073 Å
Hall symbol: -C 2yc	Cell parameters from 1589 reflections
*a* = 11.135 (7) Å	θ = 2.9–25.7°
*b* = 9.220 (6) Å	µ = 0.28 mm^−^^1^
*c* = 21.452 (15) Å	*T* = 296 K
β = 93.680 (12)°	Plate, colorless
*V* = 2198 (3) Å^3^	0.23 × 0.14 × 0.04 mm
*Z* = 4	

### Data collection

**Table:**

Bruker APEXII CCD diffractometer	1983 independent reflections
Radiation source: fine-focus sealed tube	1558 reflections with *I* > 2σ(*I*)
Graphite monochromator	*R*_int_ = 0.055
φ and ω scans	θ_max_ = 25.3°, θ_min_ = 2.9°
Absorption correction: multi-scan (*SADABS*; Sheldrick, 1996)	*h* = −13→12
*T*_min_ = 0.938, *T*_max_ = 0.989	*k* = −11→11
5135 measured reflections	*l* = −17→25

### Refinement

**Table:**

Refinement on *F*^2^	Primary atom site location: structure-invariant direct methods
Least-squares matrix: full	Secondary atom site location: difference Fourier map
*R*[*F*^2^ > 2σ(*F*^2^)] = 0.053	Hydrogen site location: inferred from neighbouring sites
*wR*(*F*^2^) = 0.150	H atoms treated by a mixture of independent and constrained refinement
*S* = 1.03	*w* = 1/[σ^2^(*F*_o_^2^) + (0.0846*P*)^2^ + 0.5056*P*] where *P* = (*F*_o_^2^ + 2*F*_c_^2^)/3
1983 reflections	(Δ/σ)_max_ = 0.001
140 parameters	Δρ_max_ = 0.32 e Å^−^^3^
1 restraint	Δρ_min_ = −0.36 e Å^−^^3^

### Special details

**Table:**

Geometry. All e.s.d.'s (except the e.s.d. in the dihedral angle between two l.s. planes) are estimated using the full covariance matrix. The cell e.s.d.'s are taken into account individually in the estimation of e.s.d.'s in distances, angles and torsion angles; correlations between e.s.d.'s in cell parameters are only used when they are defined by crystal symmetry. An approximate (isotropic) treatment of cell e.s.d.'s is used for estimating e.s.d.'s involving l.s. planes.
Refinement. Refinement of *F*^2^ against ALL reflections. The weighted *R*-factor *wR* and goodness of fit *S* are based on *F*^2^, conventional *R*-factors *R* are based on *F*, with *F* set to zero for negative *F*^2^. The threshold expression of *F*^2^ > σ(*F*^2^) is used only for calculating *R*-factors(gt) *etc*. and is not relevant to the choice of reflections for refinement. *R*-factors based on *F*^2^ are statistically about twice as large as those based on *F*, and *R*- factors based on ALL data will be even larger.

### Fractional atomic coordinates and isotropic or equivalent isotropic displacement parameters (Å^2^)

**Table:**

	*x*	*y*	*z*	*U*_iso_*/*U*_eq_	
S1	0.51163 (5)	0.03617 (7)	0.64470 (3)	0.0440 (3)	
O1	0.48265 (19)	−0.0655 (2)	0.59612 (9)	0.0590 (6)	
O2	0.41953 (16)	0.0823 (2)	0.68398 (9)	0.0561 (5)	
O3	0.87638 (18)	0.0318 (2)	0.72055 (10)	0.0567 (6)	
N1	0.6163 (2)	−0.0329 (2)	0.69058 (11)	0.0464 (6)	
H1	0.614 (3)	0.002 (3)	0.7255 (9)	0.056*	
C1	0.7115 (3)	0.5726 (4)	0.52722 (19)	0.0769 (10)	
H1A	0.7314	0.6430	0.5592	0.115*	
H1B	0.6528	0.6126	0.4973	0.115*	
H1C	0.7827	0.5473	0.5066	0.115*	
C2	0.6609 (2)	0.4389 (3)	0.55625 (15)	0.0518 (7)	
C3	0.6610 (2)	0.4248 (3)	0.62001 (15)	0.0533 (7)	
H3	0.6933	0.4992	0.6452	0.064*	
C4	0.6151 (2)	0.3047 (3)	0.64781 (13)	0.0488 (7)	
H4	0.6153	0.2980	0.6911	0.059*	
C5	0.5681 (2)	0.1927 (3)	0.60972 (12)	0.0398 (6)	
C6	0.5678 (2)	0.2038 (3)	0.54560 (13)	0.0512 (7)	
H6	0.5374	0.1288	0.5202	0.061*	
C7	0.6131 (3)	0.3270 (3)	0.51958 (14)	0.0594 (8)	
H7	0.6116	0.3352	0.4763	0.071*	
C8	0.7215 (3)	−0.1019 (3)	0.66646 (15)	0.0578 (8)	
H8A	0.6986	−0.1426	0.6257	0.069*	
H8B	0.7461	−0.1816	0.6939	0.069*	
C9	0.8272 (3)	−0.0037 (4)	0.66034 (14)	0.0566 (8)	
H9A	0.8873	−0.0518	0.6369	0.068*	
H9B	0.8019	0.0839	0.6381	0.068*	
C10	0.9623 (3)	0.1465 (3)	0.72024 (14)	0.0570 (8)	
H10A	0.9210	0.2387	0.7150	0.068*	
H10B	1.0129	0.1338	0.6855	0.068*	

### Atomic displacement parameters (Å^2^)

**Table:**

	*U*^11^	*U*^22^	*U*^33^	*U*^12^	*U*^13^	*U*^23^
S1	0.0341 (4)	0.0516 (4)	0.0450 (4)	−0.0066 (3)	−0.0080 (3)	0.0044 (3)
O1	0.0642 (13)	0.0572 (11)	0.0529 (12)	−0.0192 (10)	−0.0164 (10)	−0.0014 (9)
O2	0.0341 (10)	0.0774 (13)	0.0567 (12)	−0.0015 (9)	0.0011 (9)	0.0128 (10)
O3	0.0480 (11)	0.0616 (12)	0.0595 (13)	−0.0071 (9)	−0.0041 (10)	0.0074 (10)
N1	0.0402 (12)	0.0549 (14)	0.0428 (13)	0.0016 (10)	−0.0072 (11)	−0.0002 (10)
C1	0.067 (2)	0.0605 (18)	0.102 (3)	−0.0117 (17)	−0.004 (2)	0.0204 (19)
C2	0.0369 (14)	0.0502 (15)	0.068 (2)	0.0001 (12)	−0.0021 (14)	0.0101 (14)
C3	0.0412 (14)	0.0467 (14)	0.071 (2)	−0.0039 (12)	−0.0036 (14)	−0.0096 (14)
C4	0.0417 (14)	0.0558 (15)	0.0478 (16)	−0.0024 (12)	−0.0043 (12)	−0.0049 (13)
C5	0.0296 (12)	0.0452 (13)	0.0440 (14)	0.0015 (10)	−0.0033 (11)	0.0013 (11)
C6	0.0518 (16)	0.0545 (15)	0.0463 (16)	−0.0087 (13)	−0.0055 (13)	−0.0009 (13)
C7	0.0644 (19)	0.0631 (18)	0.0499 (17)	−0.0079 (15)	−0.0023 (15)	0.0103 (14)
C8	0.0500 (16)	0.0562 (16)	0.0658 (19)	0.0092 (13)	−0.0086 (15)	−0.0111 (15)
C9	0.0423 (15)	0.0745 (19)	0.0526 (18)	0.0121 (14)	−0.0004 (14)	−0.0021 (15)
C10	0.0504 (16)	0.0494 (15)	0.071 (2)	0.0000 (13)	0.0026 (14)	0.0071 (14)

### Geometric parameters (Å, º)

**Table:**

S1—O1	1.423 (2)	C3—H3	0.9300
S1—O2	1.433 (2)	C4—C5	1.398 (4)
S1—N1	1.608 (2)	C4—H4	0.9300
S1—C5	1.761 (3)	C5—C6	1.379 (4)
O3—C9	1.409 (3)	C6—C7	1.376 (4)
O3—C10	1.426 (3)	C6—H6	0.9300
N1—C8	1.457 (4)	C7—H7	0.9300
N1—H1	0.816 (17)	C8—C9	1.497 (4)
C1—C2	1.507 (4)	C8—H8A	0.9700
C1—H1A	0.9600	C8—H8B	0.9700
C1—H1B	0.9600	C9—H9A	0.9700
C1—H1C	0.9600	C9—H9B	0.9700
C2—C3	1.374 (4)	C10—C10^i^	1.482 (6)
C2—C7	1.383 (4)	C10—H10A	0.9700
C3—C4	1.372 (4)	C10—H10B	0.9700
			
O1—S1—O2	119.26 (13)	C6—C5—S1	120.5 (2)
O1—S1—N1	108.00 (13)	C4—C5—S1	119.1 (2)
O2—S1—N1	106.01 (13)	C7—C6—C5	119.2 (3)
O1—S1—C5	107.33 (13)	C7—C6—H6	120.4
O2—S1—C5	107.22 (13)	C5—C6—H6	120.4
N1—S1—C5	108.68 (12)	C6—C7—C2	121.5 (3)
C9—O3—C10	112.9 (2)	C6—C7—H7	119.2
C8—N1—S1	121.6 (2)	C2—C7—H7	119.2
C8—N1—H1	124 (2)	N1—C8—C9	114.9 (2)
S1—N1—H1	110 (2)	N1—C8—H8A	108.5
C2—C1—H1A	109.5	C9—C8—H8A	108.5
C2—C1—H1B	109.5	N1—C8—H8B	108.5
H1A—C1—H1B	109.5	C9—C8—H8B	108.5
C2—C1—H1C	109.5	H8A—C8—H8B	107.5
H1A—C1—H1C	109.5	O3—C9—C8	108.8 (2)
H1B—C1—H1C	109.5	O3—C9—H9A	109.9
C3—C2—C7	118.2 (3)	C8—C9—H9A	109.9
C3—C2—C1	120.8 (3)	O3—C9—H9B	109.9
C7—C2—C1	121.0 (3)	C8—C9—H9B	109.9
C4—C3—C2	122.2 (3)	H9A—C9—H9B	108.3
C4—C3—H3	118.9	O3—C10—C10^i^	109.8 (2)
C2—C3—H3	118.9	O3—C10—H10A	109.7
C3—C4—C5	118.6 (3)	C10^i^—C10—H10A	109.7
C3—C4—H4	120.7	O3—C10—H10B	109.7
C5—C4—H4	120.7	C10^i^—C10—H10B	109.7
C6—C5—C4	120.3 (2)	H10A—C10—H10B	108.2

Symmetry code: (i) −*x*+2, *y*, −*z*+3/2.

### Hydrogen-bond geometry (Å, º)

**Table:**

*D*—H···*A*	*D*—H	H···*A*	*D*···*A*	*D*—H···*A*
N1—H1···O2^ii^	0.82 (2)	2.14 (2)	2.944 (3)	171 (3)
C6—H6···O1^iii^	0.93	2.56	3.311 (4)	138

Symmetry codes: (ii) −*x*+1, *y*, −*z*+3/2; (iii) −*x*+1, −*y*, −*z*+1.
